# Spontaneous Pneumomediastinum, Pneumoperitoneum, and Subcutaneous Emphysema beyond the Inguinal Ligament Secondary to Inversion Maneuvers

**DOI:** 10.1155/2022/7054146

**Published:** 2022-06-25

**Authors:** Keegan Plowman, David Lindner, Jose Ruben Valle-Giler

**Affiliations:** ^1^NCH Healthcare System, 311 9th Street North, Naples, FL, USA 34102; ^2^Division of Pulmonary Critical Care Medicine and Associate Program Director of NCH Pulmonary Critical Care Fellowship, NCH Healthcare System, 311 9th Street North, Naples, FL, USA 34102; ^3^Division of Pulmonary Critical Care Medicine, NCH Healthcare System, 311 9th Street North, Naples, FL, USA 34102

## Abstract

Pneumomediastinum is free air within the mediastinal cavity which can spread along tissue planes leading to the accumulation of large amounts of subcutaneous emphysema. Patient is a 21-year-old male with a history of autism spectrum disorder and rhabdomyolysis who presented with diffuse “popping under the skin” and was found to have crepitus extending from his neck to his bilateral ankles. He exercises frequently and performs chin-up pullovers and will often hold his breath during this movements. He uses an inversion table but denies any valsalva maneuvers or straining while inverted. Radiological imaging demonstrated pneumomediastinum, pneumoperitoneum, and diffuse subcutaneous emphysema extending into the pelvis. Diagnosis requires a combination of history, physical exam findings, and imaging findings. Patients with spontaneous pneumomediastinum typically experience self-limited disease, and efforts should be made to minimize low yield invasive testing. Most patients can be treated on an outpatient basis after monitoring and education about potential complications.

## 1. Introduction

Pneumomediastinum is defined as free air within the mediastinal cavity [1]. Primary or spontaneous pneumomediastinum has been documented to occur in the setting of asthma exacerbation, respiratory viral infections, illicit drug use (such as cocaine or MDMA), or with maneuvers that increase intrathoracic pressure such as violent coughing, strenuous exercise, or vomiting [2]. Secondary pneumomediastinum occurs due to blunt trauma and penetrating injuries or secondary to iatrogenic causes such as surgical laparoscopy, endoscopic procedure, or intubation [1]. Commonly referred to as the Macklin effect, pneumomediastinum results from air spread from ruptured alveoli to the mediastinum along broncho vascular sheaths and subsequently along tissue planes [1, 2]. This typically leads to the accumulation of large amounts of subcutaneous emphysema. Accumulation of air within soft tissue can be self-limiting or can lead to complications.

## 2. Case Presentation

**Figure 1:**
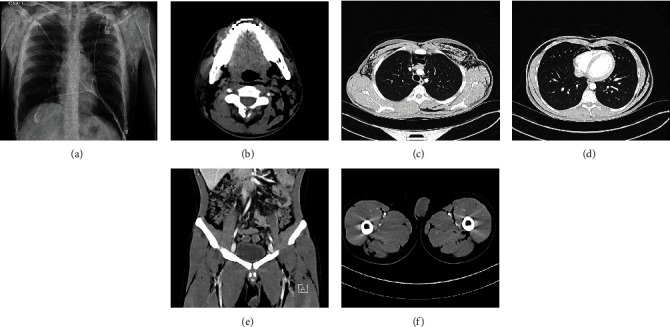
(a) Chest x-ray with pneumomediastinum with subcutaneous air in the chest wall and neck. (b) CT head with subcutaneous emphysema. (c, d) CT Chest with pneumomediastinum and extensive subcutaneous and soft tissue emphysema throughout the chest and back without signs of a pneumothorax. (e, f): CT Abdomen with pneumoperitoneum and extensive soft tissue emphysema dissecting into the pelvis and bilateral lower extremities beyond the inguinal ligament.

Patient is a 21-year-old male with a history of autism spectrum disorder and rhabdomyolysis who presented with diffuse “popping under the skin.” On evaluation, he was found to have crepitus extending from his neck to his bilateral ankles. He exercises frequently and performs chin-up pullovers with self-inversion and will often hold his breath during this movements. He also uses an inversion table, which is a device that suspends an individual in an inverted position by their ankles against gravity. He denies any valsalva maneuvers or straining while inverted. He denies any recent illnesses, straining with bowel movements, illicit drug use, asthma, pulmonary disease, scuba diving, falls, trauma, recent surgical interventions, recent endoscopies, or history of connective tissue or autoimmune disease. Labs were negative for any acute findings. COVID testing was negative. Urine drug screen was negative. Chest X-ray showed pneumomediastinum with extensive air throughout the chest wall and neck bilaterally. CT head was negative for any acute intracranial abnormalities but did demonstrate subcutaneous emphysema. CT neck showed extensive air throughout the soft tissues of the chest wall and neck, pneumomediastinum with air dissecting cephalad in the prevertebral space, and with air in the epidural space. CT chest showed pneumomediastinum and extensive subcutaneous and soft tissue emphysema throughout the chest and back without signs of a pneumothorax. CT abdomen and pelvis showed extensive soft tissue subcutaneous emphysema below the inguinal ligament into the scrotum, mild pneumoperitoneum, and mild extradural air in the lumbosacral spine. The extensive subcutaneous emphysema can be visualized in Figure 1. Gastrografin esophagram was performed which was negative for perforation. Transthoracic echocardiogram showed ejection fraction 55-60% without signs of pericardial effusion or pneumopericardium. He was monitored in the intensive care unit but remained hemodynamically stable with oxygen saturation in the high nineties on room air. The patient was discharged home with plans for repeat radiological imaging to monitor for resolution of pneumomediastinum, pneumoperitoneum, and subcutaneous emphysema.

## 3. Discussion

The patient suffered a spontaneous episode of pneumomediastinum secondary to repeat valsalva maneuvers in the setting of intense physical exercise while inverted. He developed diffuse subcutaneous emphysema and pneumoperitoneum secondary to inversion maneuvers which allowed air dissection along the spine, the pelvis, and into the bilateral lower extremities. This was easily diagnosed with radiological imaging, and the patient was managed conservatively.

Typically, subcutaneous emphysema does not traverse into the lower extremities beyond the inguinal ligaments. There is one case reported in the literature of a patient developing diffuse subcutaneous emphysema extending into the bilateral lower extremities. This occurred in a patient who was found hanging from his ankles below a bridge for an unknown amount of time [3]. This was thought to occur secondary to air dissection along tissue planes with tracking into the lower extremities due to the inverted position of the patient. As novel therapies, such as inversion tables, gain in popularity among athletes, this is an important complication to be recognized by physicians.

Diagnosis requires a combination of physical exam findings and imaging though in this case, the history of inversion maneuvers played a paramount role in diagnosis. Spontaneous pneumomediastinum is a diagnosis of exclusion. Common findings include cough, dyspnea, chest pain, dysphagia, crepitus, neck pain, and Hamman's sign [2]. Chest X-ray may demonstrate typical findings of mediastinal and subcutaneous air. CT scan should be obtained when the diagnosis is unclear or to exclude underlying pulmonary pathology [4]. Bronchoscopy and thoracic CT are performed if concern for tracheal rupture [2]. If pneumoperitoneum is present, then abdominal and pelvic CT, endoscopy, or swallow studies are performed to exclude a perforated viscus [4]. The use of invasive procedural testing is best deferred due to a low yield of results unless there is a high index of suspicion [5].

Treatment focuses on supportive care as spontaneous pneumomediastinum is usually self-limiting. Patients must be monitored for complications such as massive subcutaneous emphysema causing compression of internal structures, pneumothorax, or pneumopericardium [1]. These complications can ultimately lead to hemodynamic instability and cardiopulmonary compromise. Surgical drains can be placed for therapeutic purposes to address these complications [1]. Antibiotics and steroids have historically been used but recent studies have not shown significant benefit in the absence of perforated viscus [4]. Patients with spontaneous pneumomediastinum typically experience self-limited disease, and efforts to minimize low yield invasive testing are recommended. Most patients can be treated on an outpatient basis after monitoring and education about potential complications [5]. Follow-up imaging should be performed to ensure resolution of pneumomediastinum, pneumoperitoneum, and subcutaneous emphysema.

## Data Availability

There is no required data that would need to be made available as this is a case report.
